# Low‐protein diet for chronic kidney disease: Evidence, controversies, and practical guidelines

**DOI:** 10.1111/joim.20117

**Published:** 2025-07-31

**Authors:** Denise Mafra, Isabela Brum, Natália A. Borges, Viviane O. Leal, Denis Fouque

**Affiliations:** ^1^ Graduate Program in Biological Sciences—Physiology Federal University of Rio de Janeiro (UFRJ) Rio de Janeiro Brazil; ^2^ Graduate Program in Medical Sciences Fluminense Federal University (UFF) Niterói Rio de Janeiro Brazil; ^3^ Graduate Program in Food Nutrition and Health—Institute of Nutrition State University of Rio de Janeiro (UERJ) Rio de Janeiro Brazil; ^4^ Nutrition Division Pedro Ernesto University Hospital, State University of Rio de Janeiro Rio de Janeiro Brazil; ^5^ Department of Nephrology‐Nutrition‐Dialysis Centre Hospitalier Lyon Sud, INSERM 1060, Université de Lyon Lyon France

**Keywords:** chronic kidney disease, kidney function, low‐protein diet, nutrition

## Abstract

The benefits of a low‐protein diet (LPD) in patients with altered kidney function remain controversial. Dietary intake studies are inherently complex and may present numerous biases that must be understood and controlled. Due to these challenges, the scientific evidence in this area remains limited and is subject to dispute. However, there is abundant literature showing that excessive protein intake in these patients is linked to cardiovascular issues, oxidative stress, hyperphosphatemia, bone mineral disease, metabolic acidosis, inflammation, and gut dysbiosis, contributing to kidney damage and other concurrent systemic disorders. An LPD remains a valuable recommendation for non‐dialysis chronic kidney disease (CKD) patients if age, nutritional status, and disease complications are carefully considered to ensure optimal outcomes. On the one hand, excessive protein intake may lead to the accumulation of nitrogenous waste products, thereby burdening renal function. On the other hand, overly restrictive protein consumption can lead to muscle mass loss, potentially worsening clinical outcomes and patient prognosis. This narrative review highlights the harmful impact of a high‐protein diet on kidney function, particularly for those with preexisting kidney impairment or a predisposition to CKD. It also discusses the importance of an individualized and well‐monitored protein intake strategy to balance the benefits of protein restriction with the risks of malnutrition.

## Graphical abstract

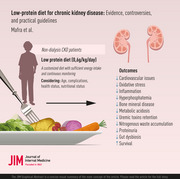

**Abbreviations**
AKI: acute kidney injury
CKD: chronic kidney disease
GFR: glomerular filtration rate
IGF‐1: insulin‐like growth factor‐1
KDIGO: Kidney Disease Improving Global Outcomes
KDOQI: Kidney Disease Outcomes Quality Initiative
KRT: kidney replacement therapy
LPD: low‐protein diet
OAT: organic anion transporters
TTDA: time‐to‐discharge‐alive from hospital

## Introduction

Regarding protein intake, the recommended daily intake for healthy adults is 0.8 g/kg/day. This amount represents the dietary protein requirement of a healthy individual (0.6 g/kg/day) plus two standard deviations (0.2 g/kg/day) needed for safe intake at the population level [1]. This recommendation of 0.8 g/kg/day is based on a reference 30‐year‐old lean man with low physical activity levels. There is ongoing debate regarding the optimal weight for estimating protein requirements across different populations, including older adults and those experiencing wasting conditions [2].

The term “low‐protein diet” (LPD) is used to describe the recommendation of 0.6 g protein/kg/day because it is 25% below the recommended amount (0.8 g/kg/day) [3]. According to the National Kidney Foundation, LPD is recommended for metabolically stable non‐dialysis chronic kidney disease (CKD) patients with glomerular filtration rate (GFR) < 60 mL/min/1.73 m^2^ in the absence of diabetes. To maintain stable nutritional status and optimize glycemic control, patients with diabetes may target a slightly broader protein intake of 0.6–0.8 g/kg/day [4].

Historically, despite the experimental research led by Dr. Brenner in the eighties, LPD has been the subject of discussion on the risks of predisposing to malnutrition and doubts regarding its effectiveness in preventing the progression of CKD. This led the Kidney Disease Improving Global Outcomes (KDIGO) to prudently recommend an intake of 0.8 g protein/kg/day (independently of diabetes diagnosis). Experts stress that protein intake should be adjusted to maintain nutritional status and prevent malnutrition [5]. Regardless of the final protein intake target, it is essential to consider that the average protein consumption in Western countries for adults is approximately 1.3 g/kg/day. From an epidemiological perspective, meeting the protein intake aligned with current recommendations for CKD (e.g., 0.8 g/kg/day) would typically require a reduction of around 40%.

Research shows that LPD can be safely implemented with sufficient energy intake (>30 kcal/kg/day). Thus, under adequate dietary supervision, LPD does not generally lead to malnutrition [6], and a precision medicine approach tailored to individual patient needs can be applied to optimize outcomes [4, 5, 7]. On the other hand, an excess of protein in the nephrons leads to glomerular hyperfiltration, which accelerates CKD progression [8, 9, 10]. Thus, this narrative review highlights the detrimental effects of a high‐protein diet on kidney function, particularly in individuals predisposed to CKD. It also underscores the importance of a personalized and carefully monitored protein intake plan for individuals with or at risk of CKD, aiming to balance the benefits of protein intake reduction with the potential risks of malnutrition.

## Protein: an essential nutrient

**Fig. 1:**
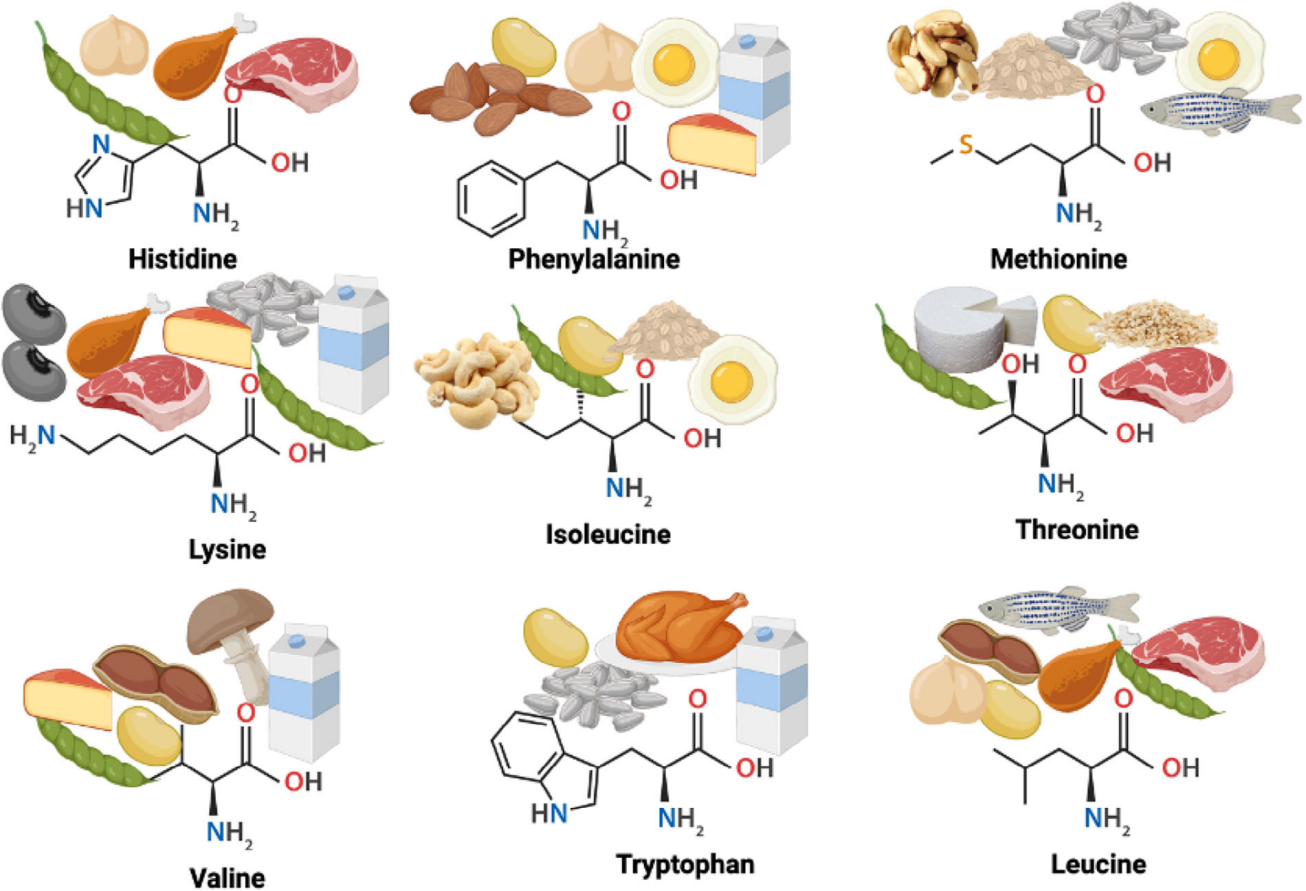
Essential amino acids and their dietary sources [13]. Created by BioRender.com

Proteins are complex organic substances composed of amino acid polymers. Among the 21 amino acids, 9 are considered essential amino acids, which are not synthesized endogenously in the human body. Therefore, they must be provided with dietary intake (Fig. 1). The chemical properties of each amino acid determine the final properties of the proteins, such as basic amino acids (histidine, lysine, and arginine) or acidic amino acids (aspartic and glutamic acid); branched‐chain amino acids such as isoleucine, leucine, and valine; aromatic amino acids phenylalanine, tryptophan, and tyrosine; and sulfur‐containing amino acids such as methionine and cysteine. Specific amino acids play crucial roles in the metabolism and physiological functions of these groups. For instance, branched‐chain amino acids are essential for protein metabolism in the skeletal muscles. Additionally, proline, tyrosine, arginine, cysteine, glutamine, and glycine are considered conditionally essential because their synthesis may be insufficient under certain conditions, such as stress or injury [11, 12].

The proportion of essential amino acids varies based on the protein source and is generally higher in animal protein sources than in plant sources. The essential amino acid content of animal‐sourced foods ranges from 43% for whey, 32% for eggs, 39% for milk, 38% for meat, and 34% for casein. In plant sources such as wheat, lupin beans, and oats, the essential amino acid content is approximately 21% [14]. Therefore, balancing amino acid intake can be achieved by combining different plant protein sources or plant and animal proteins [15]. Additionally, assessing protein quality in terms of digestibility is crucial for promoting and enhancing health, particularly in individuals with protein‐energy malnutrition. However, even the scoring systems created for protein quality assessment are limited because, among other factors, they do not adequately recognize the increased digestibility associated with commonly consumed heat‐treated and processed foods [16].

## Protein overload and kidney function

There has been a notable increase in the popularity of high‐protein diets, primarily driven by their promotion for weight loss, blood glucose control, and bodybuilding. Therefore, it is crucial to inform professionals and the general public about the effects of proteins on long‐term kidney function [9, 10]. Kidneys play a pivotal role in metabolism by breaking down and excreting proteins. Healthy individuals may not suffer from the adverse effects of a high‐protein diet. However, those with moderately reduced kidney function (e.g., GFR < 60 mL/min) or a higher risk of CKD (diabetes, obesity, and high blood pressure) may be more susceptible to harmful effects. In at‐risk individuals, excessive protein intake can lead to glomerular overload and hyperfiltration, which increases the levels of toxic metabolites in the blood that are typically cleared by healthy kidneys [17, 18].

A diet containing more than 1.5 g of protein/kg/day or a protein content exceeding 25% of the total energy of the diet may be classified as a high‐protein diet [10]. Since 1928, reports have indicated that, in animals, including both healthy and nephrectomized models, the administration of oral or intravenous amino acids can increase blood flow to the kidneys, potentially leading to long‐term renal damage [19, 20]. A high concentration of amino acids in the bloodstream due to excessive protein intake stimulates proximal tubular reabsorption of sodium, activating the renin–angiotensin–aldosterone system, which serves as a sensor for sodium concentration in the distal tubule. When this system is activated, angiotensin II is produced, which promotes efferent arteriolar vasoconstriction and increases renal blood flow, glomerular hyperfiltration, podocyte damage, and proteinuria, particularly in experimental models. Vasodilation of the preglomerular arterioles aids in the excretion of nitrogen compounds [9, 10, 17, 21, 22].

**Fig. 2:**
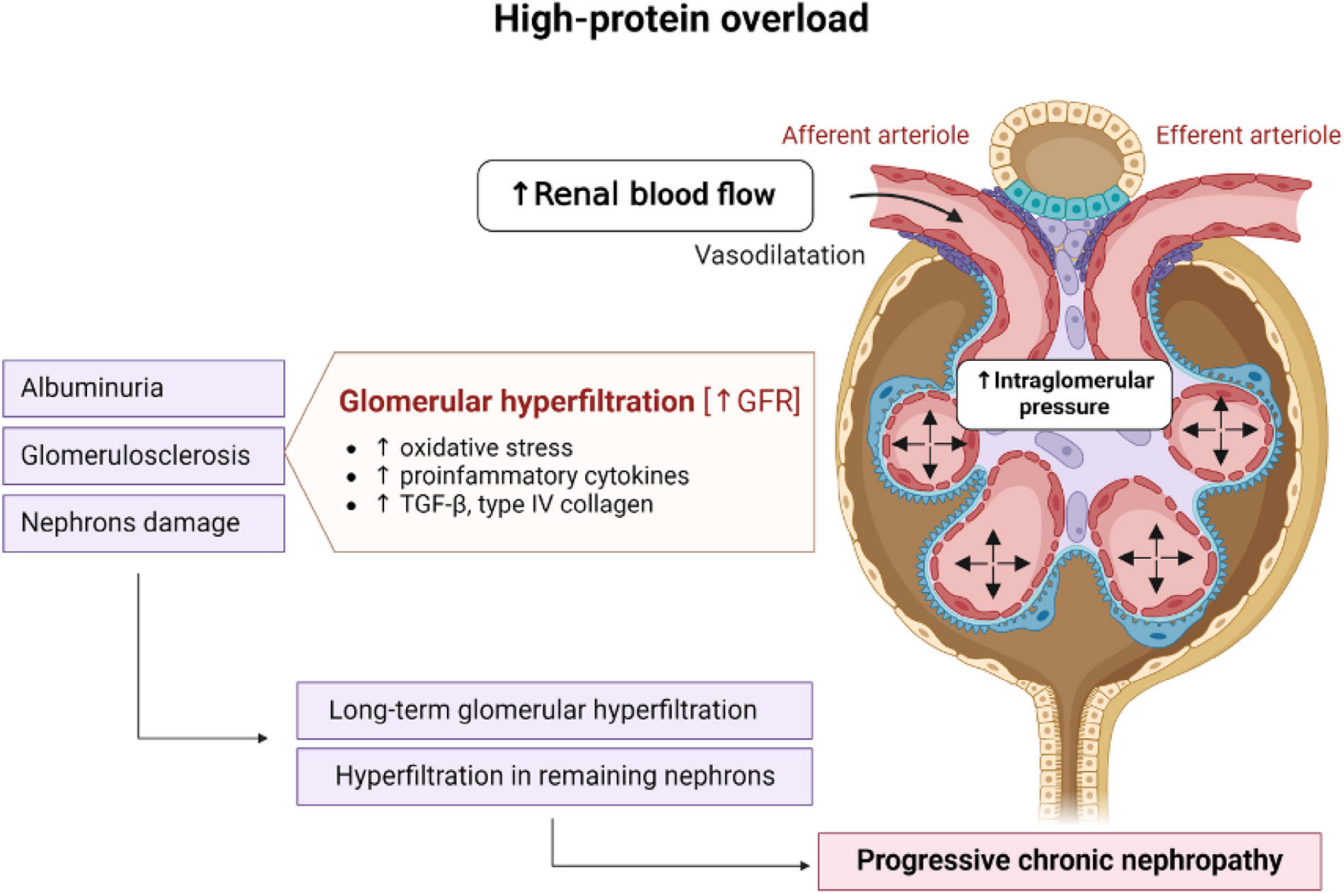
Effects of high protein intake on nephrons. High protein overload can lead to vasodilation of the afferent arteriole, increasing renal blood flow and intraglomerular pressure, ultimately resulting in glomerular hyperfiltration. This hyperfiltration elevates oxygen consumption, which may heighten oxidative stress and trigger the upregulation of pro‐inflammatory cytokines and profibrotic factors. Over time, this process can result in albuminuria, glomerular and tubular damage, and an increased burden on the remaining nephrons. Consequently, hyperfiltration may adversely impact kidney health over time, particularly in individuals at risk for or already affected by CKD. TGF‐β, transforming growth factor‐β. Source: Created by BioRender.com.

Evidence from animal models of salt‐sensitive hypertension, as well as humans without CKD, suggests that high protein intake modifies endocrine markers, elevates glucagon levels, insulin‐like growth factor‐1 (IGF‐1), and neurohormonal responses in the kidneys, eventually promoting kidney hypertrophy [23, 24] (Fig. 2). The mechanisms by which high protein intake boosts IGF‐1 synthesis include the activation of the mammalian target of rapamycin, primarily through the action of leucine. Additionally, high protein intake stimulates growth hormone secretion, further promoting IGF‐1 synthesis via the Janus kinase/signal transducer and activator of transcription (JAK‐STAT) pathway in the liver. Simultaneously, insulin secretion triggered by protein intake synergistically supports IGF‐1 synthesis. Additionally, elevated albumin excretion caused by high intraglomerular pressure hinders podocyte regeneration by promoting apoptosis in the renal tubules [17, 21, 22].

It remains controversial whether the renal‐damaging effect of excess protein consumption is similar in people with normal kidney function and without a predisposition to kidney disorders, such as diabetes or systemic high blood pressure. A large‐scale prospective cohort study evaluated the impact of protein intake on GFR over 11 years in 1624 women. This study demonstrated that the total protein intake and its subtypes (non‐dairy animals, dairy, and vegetables) in women with normal renal function were not associated with reduced GFR. However, in women with renal dysfunction (GFR < 55 mL/min/1.73 m^2^), the highest quintile of total protein intake was associated with a greater decline in GFR. Additionally, a significant association was observed between the intake of non‐dairy animal proteins and changes in the GFR [25]. This study had some limitations, particularly in measuring protein intake, which was assessed using a semiquantitative food frequency questionnaire. Although this method was validated and the authors made adjustments for measurement error, actual consumption may not have been fully captured due to the reliance on average values and participant recall. Moreover, GFR was estimated using two formulas (Modification of Diet in Renal Disease [MDRD] and Cockcroft‐Gault) rather than the gold standard method.

Esmeijer et al. [26] investigated the association between dietary protein intake and kidney function decline in 2255 patients post‐myocardial infarction over a 41‐month follow‐up period. They observed that patients consuming ≥1.2 g/kg/day of protein experienced a kidney function decline twice as fast as those consuming <0.8 g/kg/day. As this was an observational study, causal relationships cannot be confirmed, and residual confounding factors must be recognized, especially because protein intake is part of an overall dietary pattern rather than an isolated factor. Furthermore, the study did not assess the key markers of CKD progression (e.g., proteinuria), and the food frequency questionnaire used for nutritional data can lead to both underestimation and overestimation owing to its inherent limitations. Finally, because the study was conducted only at baseline, variations in dietary intake over time were not recorded.

Jhee et al. [27] evaluated 9226 healthy adult participants from the Korean Genome and Epidemiology Study (KoGES), a community‐based cohort study conducted from 2001 to 2014. They found that individuals with the highest protein intake (1.7 ± 0.6 g/kg/day) had a 3.5‐fold greater likelihood of kidney hyperfiltration and a faster decline in renal function than those with a lower protein intake (0.6 ± 0.1 g/kg/day). In this study, no data were obtained regarding the type of protein consumed by the participants, which could have influenced renal hyperfiltration and clinical conditions.

A Brazilian cross‐sectional study involving 454 non‐dialysis patients with stage 3 CKD found an inverse association between protein intake assessed at baseline using a food frequency questionnaire and eGFR [28]. Notably, the study also reported that patients consumed less energy (median of 25 kcal/kg/day) and more protein (median of 1.1 g/kg/day) than recommended, underscoring the challenges in adhering to current nutritional guidelines in this population. Additionally, there is conflicting evidence and a need for further research on the long‐term effects of high‐protein intake and its potential impact on kidney function [22].

Carballo‐Casla et al. [29] analyzed data from three retrospective cohorts and examined the relationship between protein intake and 10‐year all‐cause mortality in older adults with stages 1–3b CKD. The study suggested that higher protein intake from animal and plant sources was associated with lower mortality in older adults (≥75 years old). However, the implications of the findings remain unclear. Protein‐energy wasting, characterized by the loss of muscle mass and fat, is a common concern in older patients with CKD, and its presence can exacerbate patient mortality. There was no information on body composition, such as sarcopenia or lean body mass, which could have influenced mortality. Moreover, the methods used to assess dietary intake also varied (interviewer‐administered, validated electronic nutritional history, and self‐administered, semi‐quantitative, validated food frequency questionnaire), which may affect the reliability of protein intake assessment.

Furthermore, although the authors used current body weight in the primary analyses and ideal body weight in the sensitivity analysis with similar associations, the weight to which protein intake was normalized (current, desirable, or adjusted) is a key consideration. In cases of excess weight or malnutrition, the weight used to determine protein and energy requirements must be adjusted according to the treatment goals [4]. Therefore, comparing spontaneous current protein intake (which can be influenced by other comorbidities, such as anorexia and socioeconomic status), normalized to the actual weight of patients with altered nutritional status, may not accurately correspond to what would have been prescribed based on dietary guidelines. Another limitation was the lack of information on nephroprotective medications.

Cohort studies offer valuable insights by showing associations over time. However, applying these findings directly to clinical practice can be risky for patients with CKD, because potential confounding factors and biases can compromise the validity of the results. Recently, Mitch et al. [30] reported that the findings observed by Carballo‐Casla et al. [29] emphasized the need for a more personalized approach to protein prescriptions. With emerging therapies, such as sodium‐glucose cotransporter two inhibitors and glucagon‐like peptide‐1 receptor antagonists, evaluating protein restriction in the context of these treatments is crucial. Future research should focus on well‐designed prospective studies to better understand the long‐term effects of protein intake on kidney function and overall health. Thus, several factors may challenge the interpretation of the Carballo‐Casla et al. study as a definitive shift in dietary recommendations for older adults with mild to moderate CKD.

## Protein overload and metabolic derangements in CKD

High protein intake in non‐dialysis patients with CKD leads to complications beyond renal damage. The accumulation of serum nitrogen compounds, such as urea, is associated with cardiovascular disease, oxidative stress, inflammation, gut dysbiosis, and endothelial dysfunction [31]. Notably, proteins are metabolized into amino acids, and the excess, which cannot be stored, is eliminated by the kidneys as nitrogenous waste. With loss of renal function, this excess accumulates in the blood and can lead to uremic syndrome [32].

Furthermore, high protein intake contributes to more significant acid generation. Consequently, reduced acid excretion and lower renal bicarbonate production in patients with CKD predispose them to acidosis, thereby inducing the production of aldosterone, angiotensin II, and endothelin‐1, which promote renal fibrosis and increase inflammation. Metabolic acidosis also negatively affects bone mineralization by increasing parathyroid hormone (PTH) production and suppressing collagen production by osteoblasts, resulting in calcium withdrawal from the bones and increased urinary excretion. The acidic environment activates the adenosine triphosphate (ATP)‐dependent ubiquitin‐proteasome pathway, promoting muscle proteolysis and impairing hepatic albumin synthesis. It should also be emphasized that high protein intake (dairy products, meat, fish, and grains) is associated with increased phosphorus intake because foods rich in protein are essential sources of phosphorus. Serum phosphorus accumulation is associated with increased mortality from cardiovascular disease, likely due to the enhanced expression of fibroblast growth factor‐23, which promotes vascular calcification. Notably, inorganic phosphorus‐based additives, found in ultraprocessed foods regardless of protein content, are highly bioavailable and account for 10%–30% of dietary phosphorus intake. In addition, the increased production of uremic toxins from amino acids in the intestine may promote or contribute to gut dysbiosis [10, 21, 33, 34, 35, 36].

**Fig. 3:**
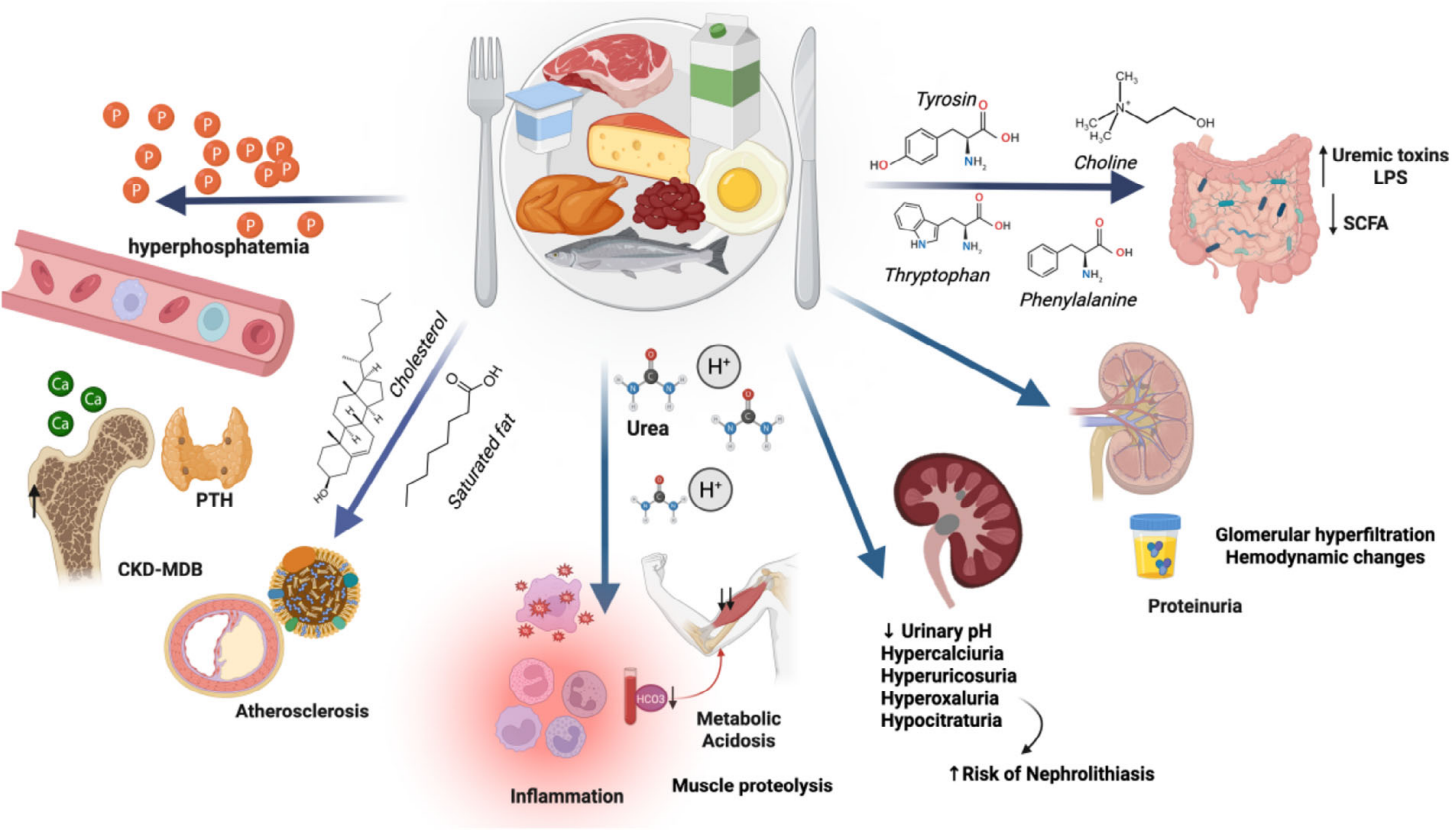
**Physiological and metabolic effects of protein overload (mainly animal protein) in CKD**. Potential implications of high‐protein diets include hyperphosphatemia and mineral and bone disorders; dyslipidemia with consequent increased risk of atherosclerosis and cardiovascular complications; accumulation of nitrogenous waste products; acidemia, which can activate proteolytic pathways, promoting muscle protein breakdown; gut dysbiosis and endotoxemia, negatively affecting immune responses; hyperalbuminemia, increased risk of nephrolithiasis; oxidative stress; and inflammation disorders. CKD–MBD, chronic kidney disease–mineral and bone disorder; LPS, lipopolysaccharides; PTH, parathyroid hormone; SCFA, short‐chain fatty acids.Created by BioRender.com

In a recent study on 5/6 nephrectomy rats, high protein intake upregulated the expression of organic anion transporters (OAT1 and OAT3) in the kidneys, influencing the tubular secretion of indoxyl sulfate (IS) levels (a uremic toxin produced by the gut microbiota). The same authors analyzed two non‐dialysis CKD patient cohorts and demonstrated higher IS excretion in those consuming a high‐protein diet (>1.2 g/kg/day) than in those consuming an LPD (<0.8 g/kg/day). This indicates that dietary protein intake influences the production of uremic toxins through the colonic microbiota and the kidney [37]. Fig. 3 illustrates the pathophysiological processes and metabolic effects of high protein intake, primarily from animal sources, in CKD.

## Low‐protein diet in experimental models

In animal models of CKD, an LPD significantly protects the kidneys by reducing urinary protein excretion, which helps slow disease progression [38]. In mice with adenine‐induced kidney failure, a low‐aromatic amino acid diet and LPD significantly reduced kidney fibrosis, proteinuria, and inflammation. Moreover, a low‐aromatic amino acid diet reduced plasma levels of uremic toxins [39]. However, applying LPD presents challenges, particularly in reducing muscle mass and protein‐energy malnutrition, which can result in low serum albumin levels. Malnutrition contributes to oxidative stress that extends beyond the kidneys [40, 41]. Oxidative stress decreases muscle mass by stimulating the ubiquitin‐proteasome system, which promotes muscle protein degradation [40]. Reduced muscle mass is linked to mitochondrial dysfunction in CKD, facilitated by the activation of the ph66Shc pathway, which amplifies the ROS generation. This redox‐dependent process renders cells sensitive to the phosphorylation of FoxO3, a transcription factor involved in protein degradation, which negatively regulates the activation of antioxidant gene expression, thereby creating a vicious cycle [40]. Studies on LPDs in experimental models are summarized in Table 1.

**Table 1:** Low protein diet in animal experimental models with chronic kidney disease (CKD).

Reference	Intervention	Main findings
Serrano et al., 2022 [42]	Alb/TGF‐β mice received LPD + HF (13% of protein and 12% of fiber) or HPD + LF (22% of protein and 4% of fiber) for 12 weeks	LPD: ↑ CD4 + Foxp3 + Treg cells in spleen and peripheral blood ↓ CD4 + Th17 + cells in spleen and peripheral blood ↑ SCFA‐producing saccharolytic bacteria
Koh 2023 [35]; Zhu et al. 2022 [38]	5/6 nephrectomy mice received NPD (20% of the weight of feed) or LPD group (5% of the weight) or LKD group (5% of the weight, 1% of which comes from α‐ketoacid) for 8 weeks	LPD: ↓ urea ↔ creatinine ↓ Lymphocyte infiltration, renal tubular lesions ↓ Fibrosis ↓ α‐SMA, IL‐1β, and IL‐6 ↓ Indoxyl sulfate
Wang et al. 2018 [7]; Chang and Anderson 2017 [36]; Wang et al. 2018 [40]	5/6 nephrectomy rat received NPD (22% of protein) or LPD (6% of protein) or LPD + KA (5% of protein +1% of KA) for 24 weeks	LPD: ↓ creatinine, urea ↓ Albumin ↑ Mitochondrial complexes I and IV ↓ Level of the FoxO3 protein and mRNA
Liu et al., 2018 [43]; Barba et al. 2021 [39]	KKAy mice model received NPD (22% of protein) or LPD (6% of protein) or LPD + KA (5% of protein and 1% of KA) for 24 weeks	LPD: ↓ 24 h urine albumin:creatinine ↑ Serum albumin ↔ Creatinine ↓ Mesangial area and glomerular basement membrane ↓ Body weight ↔ Fasting blood glucose ↓ MDA, NT ↔ SOD
Yamada et al., 2016 [44]; Wang et al. 2018 [40]	0.3% adenine rat received NPD (CNT 1.2% phosphate and 19% protein) or HP + NPD (U‐HPiNPr group 1.2% phosphate and 19% protein) or HP + VLPD (U‐HPiNPr group 1.2% phosphate and 2.5% protein) or LP + LPD (U‐HPiNPr group 0.3% phosphate and 2.5% protein) for 6 weeks	LP + LPD: ↓ urea ↔ Proteinuria, creatinine, creatinine clearance ↓ Phosphate, FGF23, FEPi, urinary phosphate excretion ↓ Body weight, serum, hepatic fetuin‐A, urinary creatinine excretion, albumin ↑ TNF‐α ↓ Calcium and phosphate content in the aorta ↓ Runx2 and calcium/phosphate precipitates
Lauriola et al. 2024 [37]; Zhang et al. 2015 [41]	5/6 nephrectomy rat received NPD (11 g of protein/kg/day) or LPD (3 g of protein/kg/day) or LPD + KA (3 g of protein/kg/day, including 5% protein and 1% KA)	LPD: ↑ body weight, gastrocnemius muscle mass and cross‐sectional area ↓ Urinary protein ↓ LC3‐GAPDH, flurorescent JC‐1 dye ↓ Relative mtDNA copy number in cytosol ↓ ROS ↑ IL‐6, ↓ IL‐1β, IL‐18
Wang et al., 2014 [45]; Zhang et al. 2015 [41]	5/6 nephrectomy rat received NPD (22% of protein) or LPD (6% of protein) or LPD + KA (5% of protein and 1% KA) for 24 weeks	LPD: ↓ body weight, muscle wet, and muscle dry ↓ Muscle protein synthesis ↓ Albumin, urinary protein, creatinine, urea ↑ Percentage of ubiquitin‐positive areas and trypsin‐like activity ↑ Relative MFI of MAFbx and relative MF1 of MuRF1 ↑ MAFbx:GAPDH and MuRF1:GAPDH ↑ TUNEL‐positive cells, caspase‐3:GAPDH ↓ Procaspaase‐3:GAPDH ↑ Actin fragment, BAX:GAPDH, Bax:Bcl2 ratio ↓ Bcl‐2:GAPDH ↓ Wnt7a:GAPDH ratio, p‐Akt:T‐Akt ratio, p‐p70S6K:Tp70S6K ratio
Gao et al., 2011 [46]; Polzin 2011 [47]	5/6 nephrectomy rat received NPD (22% of protein) or LPD (6% of protein) or LPD + KA (5% of protein and 1% KA) for 6 months	LPD: ↓ body weight ↓ Urinary protein, urea ↔ Creatinine ↓ Glomerular sclerosis index, tubulointerstitial fibrosis score ↓ Glomerular staining score of fibronectin ↓ TGF‐β, fibronectin, type I collagen, and type III collagen ↓ MCP‐1, CXCL‐1
Gao et al., 2010 [48]; IRIS [49]	5/6 nephrectomy rat received NPD (22% of protein) or LPD (6% of protein) or LPD + KA (5% of protein and 1% KA) for 24 weeks	LPD: ↓ body weight ↔ Albumin, creatinine ↓ Urea, urinary protein ↓ Glomerular sclerosis index, tubulointerstitial fibrosis score ↑ Tubulointerstitial fibronectin staining score and tubulointerstitial collagen I staining score
Cervenka and Heller, 1996 [50]; Scherk et al. 2016 [51]	5/6 nephrectomy rat received NPD (23% of protein) or NPD + enalapril or LPD (6% of protein) or LPD + enalapril for 8 weeks	↔ Creatinine, urea, creatinine clearance, and proteinuria ↔ Sodium, potassium, and urea excretion ↔ Body weight

Abbreviations: α‐SMA, alpha smooth muscle actin; Alb, albumin; CD4, cluster of differentiation 4; CXCL‐1, chemokine ligand‐1; FEPi, fractional excretion of Pi; FGF‐23, fibroblast growth factor 23; FoxO3, forkhead box P3; GAPDH, glyceraldehyde‐3‐phosphate dehydrogenase; HF, high fiber; HP, high phosphate; HPD, high‐protein diet; IL‐18, interleukin‐18; IL‐1β, interleukin‐1 β; IL‐6, interleukin‐6; KA, compound α‐ketoacid; LC3, light chain 3; LF, low fiber; LKD, α‐ketoacid diet; LP, low phosphate; LPD, low‐protein diet; MAFbx, muscle atrophy F‐box; MCP‐1, monocyte chemotactic protein‐1; MDA, malondialdehyde; MFI, mean fluorescence intensity; MuRF1, muscle ring finger‐1; NPD, normal protein diet; NT, nitrotyrosine; ROS, reactive oxygen species; Runx2, runt‐related transcription factor 2; SOD, superoxide dismutase; TGF‐β, transforming growth factor‐β; Th17, T helper 17 cells; TNF‐α, tumor necrosis factor; Treg, T regulatory cells; TUNEL, terminal dUTP nick end labeling; VLPD, very low‐protein diet.

Insights from experimental animal models have also provided information on dietary interventions for pets. CKD progression in dogs and cats involves the gradual loss of nephrons, primarily due to infections, immune diseases, and toxins that impair glomerular filtration, leading to toxin accumulation in the blood. Unlike humans, where glomerular diseases are common due to hypertension and diabetes, dogs also present with glomerular diseases but more frequently experience secondary tubular diseases. Tubulointerstitial nephritis is more prevalent in cats, whereas glomerular damage is less common, highlighting species‐specific differences in CKD progression [47]. Clinical signs, including anorexia, polyuria, polydipsia, and malnutrition, become evident after >70% nephron loss [49].

Renal diets typically include reduced levels of phosphorus, sodium, and protein, as well as increased amounts of B vitamins, soluble fiber, omega‐3 fatty acids, and antioxidants [51]. It is essential to note that phosphorus restriction has the most significant impact on preserving renal function in pets [52, 53, 54]. Early evidence from nephrectomized cats demonstrated that a low‐phosphorus diet reduced renal fibrosis compared with a higher phosphorus intake [55]. A renal diet appears to enhance the survival of these animals [53, 56] and stabilize their kidney function [57]. As is the case in humans, LPDs are controversial because inadequate protein intake can affect lean body mass. Thus, the optimal protein intake values remain undefined. Although renal diets are advantageous, particularly in advanced CKD, prospective studies assessing their effects on early stage CKD are scarce.

## Low‐protein diet for non‐dialysis CKD patients: current evidence

Protein restriction in the diet of patients with CKD was described more than 140 years ago as an essential strategy for delaying disease progression [58]. The first physician‐scientist to advocate for an LPD was Dr. Semmola in 1850, followed by Dr. Beale, who in 1869 recommended an LPD for non‐dialysis patients with CKD. In 1918, Dr. Volhard made a significant contribution to renal nutritional therapy with the introduction of a low‐protein, normal‐calorie vegetarian diet, observing a decrease in urea nitrogen, an improvement in symptoms, and an increase in patient survival [59]. In 1934, Kempner et al. administered a low‐protein, low‐fat, and low‐sodium diet to patients with CKD and reported decreased blood pressure [59]. In the 1960s, Italian researchers Giordano and Giovannetti showed that an LPD could improve uremic symptoms, delay dialysis onset, positively influence the quality of life, and reduce patient mortality [59]. Epstein et al. [60] hypothesized that reduced renal mass leads to hemodynamic changes in the remaining nephrons, contributing to further progressive deterioration of renal function through increased renal blood flow and GFR. They also observed that dietary protein restriction at the onset of the disease minimized adaptive changes, thus slowing its progression by preserving the workload of the surviving nephrons.

Rosman et al. [61], in a randomized trial involving 228 non‐dialysis patients with CKD, observed that moderate dietary protein intake slowed CKD progression. They also observed that in 149 patients monitored for at least 18 months, those adhering to a protein‐restricted diet experienced decreased serum urea, phosphate levels, and proteinuria. Moreover, critiques regarding creatinine as an outcome measure and the subjective assessment of LPD acceptance, where one‐third of patients rated it as “bad” at 3 and 6 months, do not discredit its use [62]. These criticisms may not be considered solid evidence of the benefits of the LPD approach. In 1989, Ihle et al. [63] conducted an 18‐month, prospective, randomized trial involving 64 non‐dialysis patients with CKD. The participants were randomly assigned to either a standard diet or an isocaloric diet with restricted protein (providing 0.4 g/kg/day). CKD stage 5 occurred in 27% of patients on the standard diet compared to only 6% in the protein‐restricted group, indicating that LPD is an effective method for slowing CKD progression. There was significant weight loss in the intervention group; however, the diet did not alter muscle mass markers such as mid‐arm circumference.

In an Italian multicenter trial, 456 patients with CKD were randomized to either an LPD (0.6 g/kg/day) or a controlled‐protein diet (1.0 g/kg/day) for up to 2 years or until renal endpoints were reached. The overall difference in renal survival was only borderline significant, with a considerable benefit observed in one subgroup. Poor compliance in the LPD group limited the intended dietary differences [64].

In 1994, the MDRD study was the largest randomized clinical trial to investigate the effects of LPD on CKD progression. In this study, 585 patients with GFR between 25 and 55 mL/min/1.73 m^2^ were assigned to usual protein intake or LPD (1.3 or 0.58 g protein/kg/day, respectively). Also, 255 patients with a GFR between 15 and 24 mL/min/1.73 m^2^ were assigned to LPD or a very LPD supplemented with ketoanalogs (0.58 or 0.28 g protein/kg/day). After a mean follow‐up of 2.2 years, the decline in GFR did not differ between the diet groups [65]. The MDRD trial remains controversial due to concerns about dietary adherence and its inability to meet primary efficacy endpoints. A secondary analysis of the MDRD study raised doubts about these inconclusive findings due to the differing progression mechanisms of several CKD etiologies, the ideal follow‐up duration, and the preference for analysis based on actual protein intake. Thus, the same authors of the original study published a comment 5 years later and concluded that “*the balance of evidence appears to be more consistent with the dietary efficacy hypothesis than with the contrary hypothesis of no beneficial effect*” [66].

Piccoli et al. [67] demonstrated, in an observational study, that LPD has additional benefits, including reducing the high cost of dialysis, avoiding dialysis in older patients, and increasing their survival. Systematic reviews suggest that LPD may benefit patients with CKD stages 4 and 5 [6, 68], as supported by recent nutrition guidelines [4]. Therefore, a fundamental approach to the management of CKD remains an LPD. However, it should be tailored to individual patient needs to optimize outcomes by considering factors such as the stage of CKD, the patient's overall health, age, nutritional status, and comorbidities [4].

Rhee et al. [68] conducted a systematic review and meta‐analysis to assess the application of LPD in the clinical management of patients with CKD. They evaluated 16 randomized clinical trials that compared a protein‐restricted diet (<0.8 g/kg/day) and a diet without protein restriction (>0.8 g/kg/day). The authors concluded that the protein‐restricted group had higher serum bicarbonate levels, lower phosphorus levels, a lower rate of progression to renal replacement therapy, and a lower rate of death from all causes, demonstrating the benefits of a protein‐restricted diet. This meta‐analysis also showed considerable heterogeneity among interventions. Additionally, these studies did not evaluate the risk of publication bias. Finally, a retrospective multicenter cohort study conducted in Japan showed that LPD (0.5 g/kg/day) over an average follow‐up period of approximately 4 years reduced all‐cause mortality and delayed the initiation of renal replacement therapy [69].

KDIGO [5, 70] defined rapid progression of CKD as a decline greater than 5 mL/min/1.73 m^2^/year. Hansen et al. [71] tested the effect of LPD (<0.6 g/kg/day) compared with usual protein intake (1 g/kg/day) in delaying CKD progression and improving survival in patients with type 1 diabetes and CKD over 4 years. The annual rate of decline in GFR was 3.8 mL/min/1.73 m^2^ in the low‐protein group and 3.9 mL/min/1.73 m^2^ in the usual protein group, with no difference between the groups. However, the authors observed that 27% of patients with average protein intake had stage CKD 5 or died compared to only 10% in the LPD group. A prospective study that followed over 1400 patients with CKD stage 3–4 for 5 years revealed that after adjusting for relevant covariates, each additional 0.1 g of protein intake per kg was associated with an increased hazard ratio (1.09 [95% confidence interval 1.04–1.14]) for kidney replacement therapy (KRT). This suggests that lower protein intake is linked to a slower progression to the final stage of CKD [72] and that any 0.1 g/kg reduction in protein intake is worth doing.

The KNOW‐CKD was a multicenter prospective cohort study conducted in Korea between 2011 and 2016. It included 1572 patients with CKD stages 1–5 and was designed to study the association between protein intake and renal function. The outcome was a GFR decline of more than 50% or a doubling of serum creatinine and initiation of dialysis. The authors concluded that protein intake alone did not determine CKD progression [73].

Corroborating these results, a meta‐analysis aimed at determining the efficacy of LPD in outcomes such as the start of dialysis or death in non‐diabetic patients began collecting data in the 2000s and was last updated in 2020. Seventeen randomized clinical trials were included, totaling 2996 participants, with a mean follow‐up period of 12–50 months. Studies comparing an LPD (0.5–0.6 g/kg) with a normal‐protein diet (≥0.8 g/kg) in patients with stage 3 CKD have shown little or no difference in achieving KRT, demonstrating that it is uncertain whether LPD compared with regular protein intake can promote benefits to patients with CKD [6]. However, patients undergoing a protein intake below 0.5 g/kg/day, supplemented with ketoanalogs, did show a significant reduction in mortality and the need to start dialysis compared to those with higher protein intakes [6].

Baragetti et al. [8] conducted a retrospective analysis of 299 patients with stage 4 CKD over 8 years. Patients were divided into three groups: controlled protein diet (0.8 g/kg), an LPD (0.6 g/kg), and an unrestricted protein diet, in which patients did not undergo nutritional monitoring. This analysis revealed a delay in the start of dialysis of almost 24 months in the LPD group compared with that in the unrestricted protein diet group. This study has limitations inherent to a retrospective analysis. Furthermore, the muscle mass was not assessed.

Despite a significant number of epidemiological and clinical trials, only a few studies have targeted GFR measures to assess the effects of LPD on renal function and CKD progression. Many of these studies also included death and the need to start dialysis or transplantation, which are reliable hard‐point events but not proper renal function assessments.

## Low‐protein diet: how much should be prescribed?

The main nutritional recommendation for non‐dialysis patients is LPD, regardless of its origin (animal or vegetable), which may reduce the rate of disease progression [4]. Nutritional recommendations for energy and protein intake in non‐dialysis patients with CKD are shown in Table 2 [4]. The KDIGO CKD guidelines recommend a protein intake of 0.8 g/kg of body weight per day for adults with CKD stages G3 to G5. However, these guidelines do not recommend a daily energy intake [5].

**Table 2:** Daily energy and protein recommendations for non‐dialysis chronic kidney disease (CKD) patients.

Recommendations	Kidney Disease Outcomes Quality Initiative (KDOQI)‐2020 [4]
Energy (kcal/kg/day)	25–35 35—for pregnant women using pre‐gestational weight, 85 kcal/day should be added in the first, 275 kcal/day in the second, and 475 kcal/day in the third trimester of pregnancy
Protein (g/kg/day)	0.55–0.60 or 0.28–0.43 with ketoanalogs to complement (0.55 and 0.60) In the presence of diabetes: 0.60–0.80

Recommendations	Kidney Disease Improving Global Outcomes (KDIGO)‐2020
Energy	Not addressed
Protein (g/kg/day)	0.80, independently of comorbidities, or 0.3–0.4 supplemented with essential amino acids or ketoanalogs up to 0.60

In the elderly population, particularly those with CKD, protein requirements may differ owing to age‐related sarcopenia, changes in protein metabolism, and the need to balance muscle preservation with kidney function. Current recommendations suggest that protein intake for healthy older adults should be 1.0–1.2 g/kg/day [2], whereas those with CKD without dialysis may require 0.6–0.8 g/kg/day, depending on disease progression and nutritional status [4]. It is essential to note that for older people with acute or chronic disease and malnutrition, the protein should be 1.2–1.5 g protein/kg body weight/day [2]. Therefore, it is crucial to emphasize the importance of precise nutrition in managing CKD because a “one‐size‐fits‐all” approach may not be suitable.

Although the Kidney Disease Outcomes Quality Initiative (KDOQI) [4] only recommends the amount of protein and does not suggest its source, there is growing evidence that a low‐protein, plant‐dominant diet reduces disease outcomes, such as cardiometabolic complications and metabolic acidosis. The predominant consumption of plant‐based proteins could also be beneficial in reducing the production of uremic toxins owing to the greater consumption of dietary fiber, which can lead to a greater intestinal output of short‐chain fatty acids and lower production of toxins by intestinal bacteria generated from essential amino acids present in foods of animal origin [74, 75]. Moreover, a diet with more vegetables and fruits has a beneficial impact on mineral bone disorders because the bioavailability of phosphorus from plant sources is lower, up to approximately 40%, owing to the presence of phytates in plants [76].

## Low‐protein diet: safety and quality of life improvement in non‐dialysis CKD patients

Studies have demonstrated the safety of LPD in non‐dialysis patients with CKD when guided by specialist professionals [77, 78]. Windahl et al. [79] evaluated the safety of prescribing an LPD (≤0.8 g/kg) in 1738 individuals over 65 years and GFR < 20 mL/min/1.73 m^2^ through the analysis of nutritional status assessed by the 7‐point subjective global analysis. No significant changes in nutritional status or mortality risk were observed in patients receiving LPD. Hahn et al. [6] reported that 514 patients, whether or not they received LPD/VLPD, did not lose weight during their study. Ihle et al. [63] observed significant weight loss in the LPD group, but albumin plasma levels and muscle mass markers were preserved. Martino et al. [80] evaluated the consumption of an LPD (≤0.6 g/kg/day) for 4–6 months in 45 patients aged over 70 years and observed that the use of this diet had no impact on sarcopenia or worsening of the body composition. Furthermore, the authors demonstrated beneficial reductions in plasma urea levels and proteinuria.

**Fig. 4:**
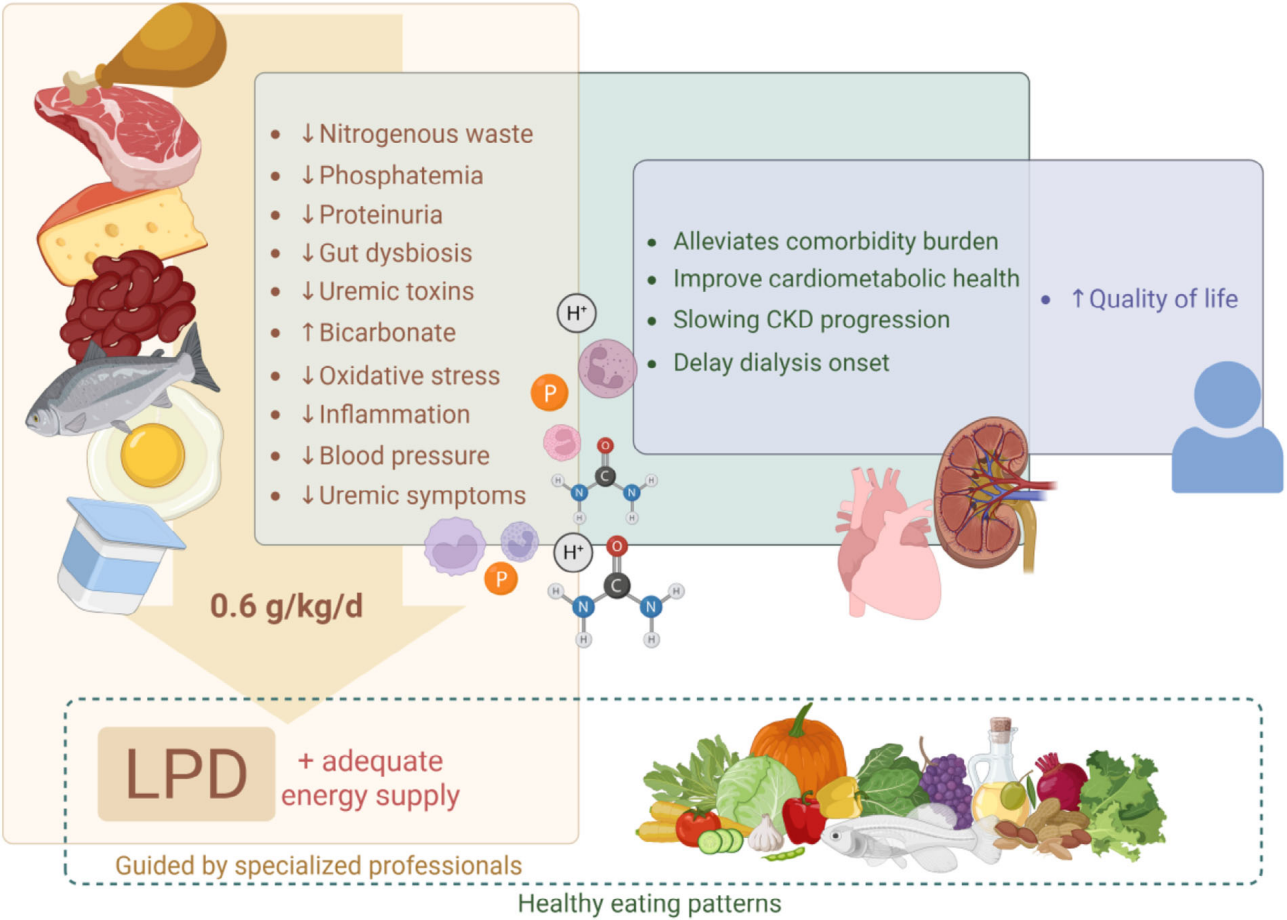
**Potential benefits of the LPD diet to CKD patients**. Molecular and metabolic changes promoted by a programmed protein restriction, guided by a specialized professional and ensuring adequate energy intake, combined with a healthy eating pattern, can contribute to the improvement of comorbidities, enhance cardiometabolic health, slow CKD progression, delay the onset of dialysis, and ultimately improve patients’ quality of life. CKD, chronic kidney disease; LPD, low‐protein diet. Created by BioRender.com

Some professionals are concerned about patients’ quality of life when prescribing LPD. However, studies have shown that a decrease in quality of life is related to comorbidities associated with CKD, disease progression, and age, regardless of the amount of dietary protein consumed [67, 81]. In addition, a significant loss of quality of life, when patients start dialysis [82], can be avoided in those receiving LPD who postpone dialysis initiation by months or years. Therefore, managing non‐dialysis CKD patients requires strategies that address CKD and its comorbidities to maintain renal function, delay the initiation of KRT, and enhance cardiometabolic health and quality of life (Fig. 4).

## Optimal protein intake in critically ill patients

In critically ill patients, protein‐energy malnutrition and muscle wasting are significant prognostic factors influenced by the endocrine‐metabolic response, which includes hypercatabolism and pronounced alterations in protein metabolism. In addition, therapeutic interventions, such as mechanical ventilation, can negatively impact this scenario. Therefore, providing protein is crucial for preserving muscle mass, maintaining physical function, and improving clinical outcomes. High protein intake has long been considered beneficial for these patients. Supported by limited evidence, a range of 1.2–2.0 g/kg actual body weight per day or more (up to 2.5 g/kg/day), depending on particular situations (e.g., obese individuals), is expected [83, 84]. In addition, the protein recommendation to patients with acute kidney injury (AKI) with acute critical illness, not on KRT, is 1.0–1.3 g/kg/day, reaching 1.5 or 1.7 g/kg/day in those on intermittent or continuous KRT, respectively [85]. However, the potential benefits of high protein intake in critically ill patients have been challenged by recent clinical trials that compared standard versus higher protein intake [83, 84, 86, 87, 88].

The EFFORT Protein study, a large multicenter trial involving mechanically ventilated adults with high nutritional risk, compared prescribing the usual dose of protein (≤1.2 g/kg/day) with high‐dose protein (≥2.2 g/kg/day). Increasing amino acid delivery beyond needs may raise blood urea nitrogen production and push patients into requiring acute dialysis and additional iatrogenic issues. High protein intakes did not improve time‐to‐discharge‐alive from hospital (TTDA) or 60‐day mortality, and patients with high‐dose protein had a higher urea concentration [86]. Additionally, in a post hoc analysis of the EFFORT study involving a subgroup of critically ill patients with AKI, protein dose was not significantly associated with the incidence of AKI and KRT or duration of KRT. However, high protein provision was associated with slower TTDA from the hospital and higher 60‐day mortality in all AKI stages (KDIGO 1–3) not receiving KRT. No higher or lower protein dosing effect was observed in AKI patients receiving KRT [89].

According to the authors, increased ureagenesis in response to high protein intake may indicate that patients with AKI and impaired muscle protein synthesis experience a metabolic burden due to excessive protein breakdown. The authors emphasize that the theoretical phases of critical illness were not considered in the EFFORT study, and the same dose was administered throughout the entire study period [86, 89]. Notably, the protein provision in the high‐dose protein group exceeded the recommended levels for critically ill patients not undergoing KRT.

In another large multicenter trial, the PRECISe study compared the effects of standard (1.3 g/kg/day) or high (2.0 g/kg/day) protein diets in mechanically ventilated critically ill adults (not including BMI <18 kg/m^2^ or AKI not on dialysis). High enteral protein provision resulted in a lower health‐related quality of life and increased TTDA compared with patients allocated to standard protein administration. Furthermore, the incidence of gastrointestinal intolerance and use of prokinetics was more significant in patients assigned to the high‐protein group. No unfavorable effects of high protein provision were observed in patients with AKI, but in this study, non‐dialysis CKD patients were not included [87].

The authors indicated that a greater supply of amino acids might have surpassed the capacity for protein synthesis, resulting in increased amino acid oxidation, the production of toxic deamination byproducts, and an uptick in urea production that could necessitate more dialysis sessions. Moreover, administering exogenous proteins during the acute phase of critical illness may hinder autophagy, when dysfunctional cellular components, including proteins, are degraded and recycled. The PRECISe and EFFORT Protein trials corroborate one another's findings, failing to support the hypothesis that high‐protein nutrition enhances clinical outcomes in critically ill patients [87].

According to recent meta‐analyses, the potential benefits of high protein or amino acid delivery compared to normal protein delivery on clinical outcomes for critically ill patients are not substantiated. Conversely, the findings suggest that higher protein diets may elevate mortality rates in critically ill patients with AKI, particularly in those who did not receive KRT [89, 90, 91]. In contrast, some results suggest higher protein delivery may attenuate muscle loss [91]. The optimal protein range for critically ill patients remains to be determined but should not exceed 1.5–1.7 g/kg/day. All the aforementioned data suggest that even in critically ill patients, where many factors could justify an increased protein intake, the adage “the more protein, the better” does not hold true. Individualized nutrition is essential for providing nutrients tailored to the patient's specific condition and the particular phase of their illness.

## Final remarks

LPD is a well‐established strategy for improving health outcomes in non‐dialysis patients with CKD. Although there is no consensus on its effectiveness in delaying CKD progression, its positive effects on phosphate metabolism, cardiovascular outcomes, intestinal dysbiosis, and metabolic acidosis are noteworthy. However, in specific clinical situations such as compromised nutritional status, catabolic diseases, cachexia, active inflammation or infection, and difficulty reaching energy goals, protein requirements must be individualized to avoid malnutrition and undesirable related effects such as hospitalization and mortality. Therefore, LPD appears to be safe and is recommended for patients with metabolically stable non‐dialysis CKD. Well‐planned dietary care can improve patients’ nutritional status in CKD stages 3–5 before dialysis, reduce CKD‐associated dysmetabolism, decrease medication use, and postpone the need for dialysis maintenance.

## Conflict of interest statement

The authors declare no conflicts of interest.

## Funding information

Conselho Nacional de Pesquisa (CNPq), Coordenação de Aperfeiçoamento de Pessoal de Nível Superior (CAPES), and Fundação de Amparo à Pesquisa do Estado do Rio de Janeiro (FAPERJ)
